# CLOSE KIN TIES AND COVID-19 OUTCOMES AMONG OLDER ADULTS IN EUROPE: THE ROLE OF COVID-19 PRECAUTIONARY BEHAVIORS

**DOI:** 10.1093/geroni/igad104.1865

**Published:** 2023-12-21

## Abstract

The purpose of this study is to examine the relationship between the availability of close kin ties (such as spouses and children) and the risk of COVID-19 contagion among Europeans who are 50 years of age or older. We also investigate the extent to which COVID-19 precautionary behaviors (such as mask-wearing, physical distancing, and frequent handwashing) mediate this relationship. Data were retrieved from the eighth wave of the Survey of Health, Ageing and Retirement in Europe (SHARE) and two waves of the SHARE Corona Survey (SCS). We used logistic regression models and the Karlson-Holm-Breen (KHB) decomposition method to analyze the data. Our preliminary findings indicate that individuals who have close family members are more likely to engage in precautionary behaviors. Furthermore, we found that engaging in such behaviors was associated with a reduced risk of COVID-19 outcomes. These initial results suggest that having close family ties may play an important role in shaping the responses of older adults to the COVID-19 pandemic. Additionally, our study highlights the importance of engaging in self-protective behaviors as a means of reducing the risk of COVID-19 outcomes among older adults.

